# Multiple ubiquitin E3 ligase genes antagonistically regulate chloroplast-associated protein degradation

**DOI:** 10.1016/j.cub.2023.01.060

**Published:** 2023-03-27

**Authors:** Sabri Mohd. Ali, Na Li, Ziad Soufi, Jinrong Yao, Errin Johnson, Qihua Ling, R. Paul Jarvis

**Affiliations:** 1 Section of Molecular Plant Biology (Department of Biology) and Department of Plant Sciences, University of Oxford, South Parks Road, Oxford OX1 3RB, UK; 2 University of Chinese Academy of Sciences, Beijing 100049, China; 3 The Sir William Dunn School of Pathology, University of Oxford, Oxford OX1 3RE, UK; 4 National Key Laboratory of Plant Molecular Genetics, CAS Centre for Excellence in Molecular Plant Sciences, Institute of Plant Physiology and Ecology, Chinese Academy of Sciences, Shanghai 200032, China

**Keywords:** CHLORAD, chloroplast, E3 ligase, plastid, ubiquitin-proteasome system

## Abstract

The chloroplast is the most prominent member of a diverse group of plant organelles called the plastids, and it is characterized by its vital role in photosynthesis.^1^^,^^2^^,^^3^ Most of the ∼3,000 different proteins in chloroplasts are synthesized in the cytosol in precursor (preprotein) form, each with a cleavable transit peptide.^4^^,^^5^^,^^6^^,^^7^^,^^8^ Preproteins are imported via translocons in the outer and inner envelope membranes of the chloroplast, termed TOC and TIC, respectively.^9^^,^^10^^,^^11^^,^^12^^,^^13^ Discovery of the chloroplast-localized ubiquitin E3 ligase SUPPRESSOR OF PPI1 LOCUS1 (SP1) demonstrated that the nucleocytosolic ubiquitin-proteasome system (UPS) targets the TOC apparatus to dynamically control protein import and chloroplast biogenesis in response to developmental and environmental cues. The relevant UPS pathway is termed chloroplast-associated protein degradation (CHLORAD).^14^^,^^15^^,^^16^ Two homologs of SP1 exist, SP1-like1 (SPL1) and SPL2, but their roles have remained obscure. Here, we show that SP1 is ubiquitous in the Viridiplantae and that SPL2 and SPL1 appeared early during the evolution of the Viridiplantae and land plants, respectively. Through genetic and biochemical analysis, we reveal that SPL1 functions as a negative regulator of SP1, potentially by interfering with its ability to catalyze ubiquitination. In contrast, SPL2, the more distantly related SP1 homolog, displays partial functional redundancy with SP1. Both SPL1 and SPL2 modify the extent of leaf senescence, like SP1, but do so in diametrically opposite ways. Thus, SPL1 and SPL2 are bona fide CHLORAD system components with negative and positive regulatory functions that allow for nuanced control of this vital proteolytic pathway.

## Highlights

• SPL1 and SPL2 are chloroplast SP1 homologs that appeared early in plant evolution
• SPL1 negatively regulates SP1, potentially interfering with its E3 ligase activity
• SPL2, the more divergent SP1 homolog, shares functional redundancy with SP1
• SPL1 and SPL2 affect leaf senescence, similar to SP1, but in diametrically opposite ways

## Graphical abstract

**Figure:**
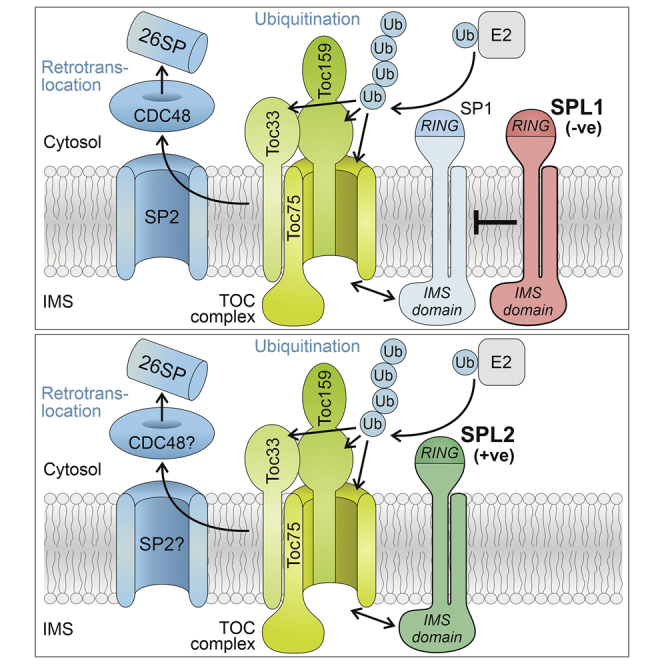

## Teaser

Mohd. Ali et al. study SPL1 and SPL2, homologs of ubiquitin E3 ligase SP1, which mediates chloroplast-associated protein degradation (CHLORAD). SPL1 negatively regulates SP1, whereas SPL2 shares redundancy with SP1. Both homologs affect leaf senescence, but in diametrically opposite ways. Thus, multiple E3 genes antagonistically regulate CHLORAD.

## Results

### The *SPL2* gene is partially redundant with *SP1*

The *SP1* gene (TAIR: At1g63900) was identified in a genetic screen for second-site suppressors of the *Arabidopsis thaliana* Toc33 knockout mutation, *plastid protein import1* (*ppi1*).^14^ The *sp1* mutation causes recovery of the chlorotic *ppi1* phenotype by improving TOC protein stability and, consequently, chloroplast preprotein import capacity and development due to disruption of the CHLORAD proteolytic pathway.^16^ Although *SPL1* (TAIR: At1g59560) and *SPL2* (TAIR: At1g54150) share amino acid sequence identity (60.3% and 20.5%, respectively) and topological similarity with SP1,^14^ their functions are poorly defined. Notwithstanding some alternative reports,^17^^,^^18^^,^^19^^,^^20^ confocal microscopy revealed chloroplast envelope localization similar to SP1 for both homologs.^14^^,^^21^ However, *SPL1* overexpression did not complement the phenotype of *sp1 ppi1* mutants, indicating that SPL1 is not redundant with SP1.^14^ Little is known about the role of *SPL2*, although its indirect involvement in drought tolerance via noncoding RNA and an adjacent drought-stress inducible transcription factor gene has been suggested.^17^ Given the lack of information concerning the functions of the *SPL* genes, we investigated the possibility that they are regulators of chloroplast biogenesis.

**Figure 1:**
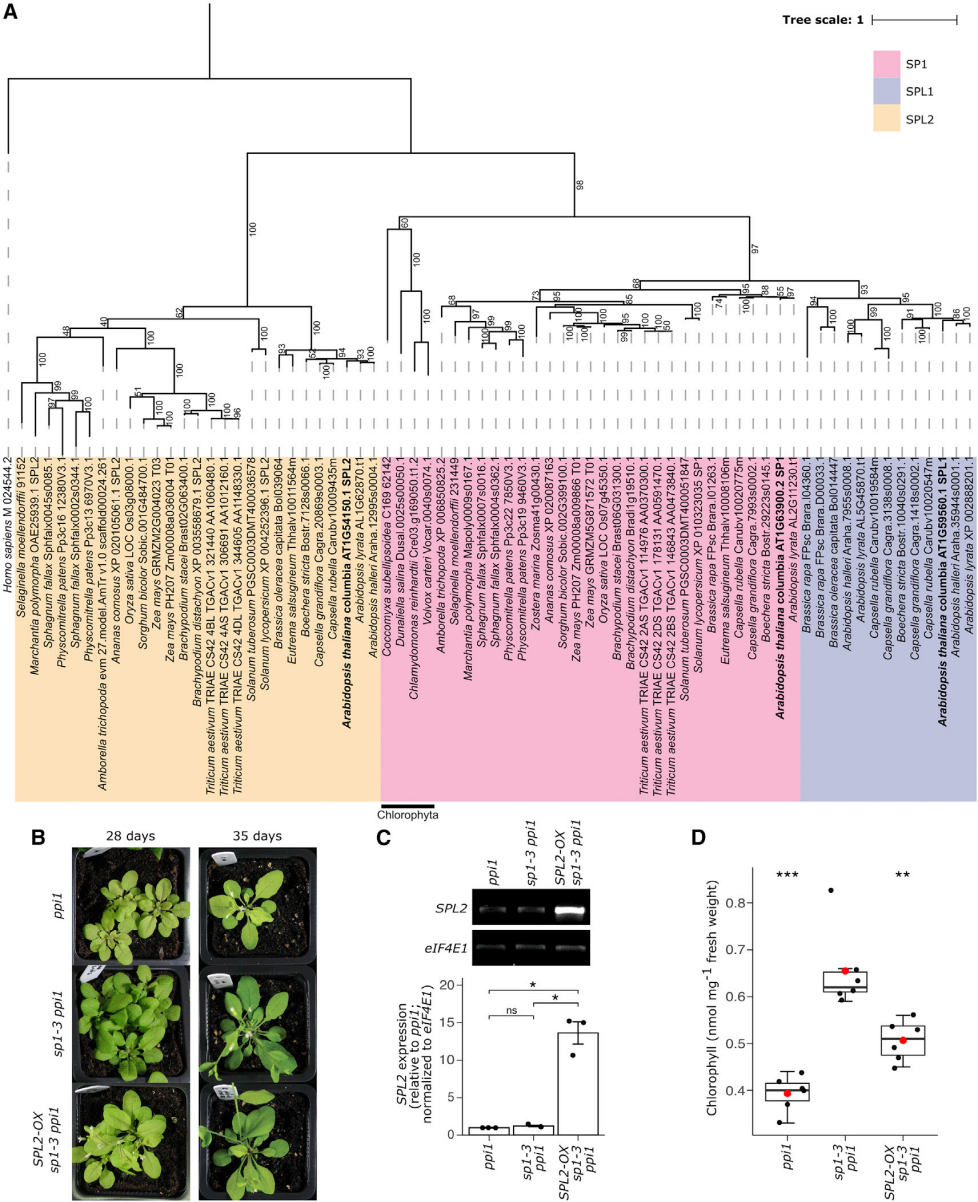
Analysis of the relationship between SP1, SPL1, and SPL2 (A) Phylogenetic analysis of SP1 and its homologs. A bootstrapped maximum likelihood evolutionary analysis of SP1 homologs in the Viridiplantae. Multiple sequence alignment of amino acid sequences retrieved from multiple databases was performed using MergeAlign and the tree was inferred using W-IQ-TREE with 1,000 ultrafast bootstrap replicates. The related human mitochondrial protein MULAN was included as an outgroup. The scale bar indicates the average number of substitutions per site, and percentage ultrafast bootstrap values are shown. The SP1, SPL1, and SPL2 clades are shaded pink, violet, and yellow, respectively. *Arabidopsis thaliana* genes are highlighted in bold text. (B–D) Analysis of the effects of *SPL2* overexpression (OX) in the *sp1 ppi1* double-mutant background. The *Arabidopsis SPL2* cDNA was cloned downstream of the constitutive cauliflower mosaic virus (CaMV) 35*S* promoter in the binary vector pB2GW7, and corresponding transgenic plants were grown on soil under standard growth conditions for 28 or 35 days before photography (B). Overexpression was confirmed by semi-quantitative RT-PCR analysis of the *SPL2-OX sp1-3 ppi1* and control plants using gene-specific primers for *SPL2* and the reference gene *eIF4E1*. The data are shown as mean ± SEM from three independent experiments (C). Plant chlorophyll contents were measured using a Konica Minolta SPAD-502 meter; data were converted to chlorophyll values as nmol per mg fresh weight (D). Measurements were made from six different individuals and presented as boxplots with the central box representing values between the first and third quartiles and the middle line representing the median value. The whiskers extend to the minimum and maximum values. The red circles indicate the means. The black circles in (C) and (D) show the individual data points. The p values were derived from Student’s t test analysis (two-tailed) of the means of the indicated genotypes (ns, not significant; ^∗^p < 0.05; ^∗∗^p < 0.01; ^∗∗∗^p < 0.001). The *sp1-3 ppi1* genotype was used as the reference group for the statistical analysis in (D). See also Figure S1.

We began by exploring the evolutionary relationship between *SP1* and its homologs. Phylogenetic analysis showed that SP1 orthologs are ubiquitous across the plant kingdom, including Chlorophyta (green algae) (Figure 1A). Branch lengths for vascular plant species in this group were notably short, indicating very low frequencies of amino acid substitution. This supports the view of SP1 as an important, highly conserved E3 ligase required for TOC regulation during the biogenesis and operation of plastids.^14^^,^^15^ Furthermore, the close relationship between SP1 and SPL1 was confirmed, as these sequences were derived from a common ancestor within the SP1 clade, right after the divergence of the Embryophyta (land plants) from the Chlorophyta. However, the number of species represented in the SPL1 subclade was greatly reduced, with only members of the Brassicaceae family of flowering plants (including the crops *Brassica oleracea* and *B. rapa*) present. This suggests that SPL1 was subsequently lost in species outside of the Brassicaceae. Branch lengths in the SPL1 subclade were longer than those in the SP1 subclade, revealing accelerated divergence of SPL1 sequences. This may indicate neofunctionalization or pseudogene formation,^22^ consistent with the non-redundancy conclusion of the earlier complementation experiment.^14^

Orthologs of SPL2 were also ubiquitous in land plants (Figure 1A). However, unlike SP1, SPL2 was not present in any of the representative green algal species, so the liverwort *Marchantia polymorpha* was the earliest diverging species with this sequence in our analysis. The SPL2 orthologs formed a monophyletic group that was derived from a common ancestor, which also gave rise to the SP1/SPL1 clade, so we infer that SPL2 was subsequently lost in green algae. Branch lengths in the SPL2 clade were more varied than those in the SP1 clade, implying different SPL2 evolution rates. This suggests greater conservation in some species and more divergence in others.

Potential SPL2 involvement in regulating the chloroplast protein import machinery, suggested by the phylogeny, was investigated by gene complementation in *Arabidopsis*. An *SPL2* overexpression (*SPL2-OX*) construct was stably transformed into *sp1-3 ppi1* plants (Figures 1B and S1A), producing an ∼14-fold increase in *SPL2* mRNA (Figure 1C). The transgenic plants were visibly paler than *sp1-3 ppi1* controls, with reduced chlorophyll content (Figures 1B, 1D, and S1A). Moreover, the transgenics contained reduced TOC protein levels, relative to *sp1-3 ppi1* control plants, indicating that SPL2 shares functional redundancy with SP1 (Figure S1B). The detected complementation was incomplete, however, as the *SPL2-OX sp1-3 ppi1* plants were still greener than *ppi1* (Figures 1B, 1D, and S1A), suggesting that the redundancy is partial.

### The *SPL2* gene regulates the chloroplast protein import machinery

Next, the involvement of *SPL1* and *SPL2* in chloroplast biogenesis was investigated genetically.^23^ To do this, we obtained T-DNA knockout mutants for *SPL1*^20^^,^^24^ (Figure S1C), and because suitable T-DNA lines were not available for *SPL2*,^17^ we generated *SPL2* knockdown (*SPL2-KD*) lines using an artificial microRNA (amiRNA) approach.^25^^,^^26^ The *SPL2-KD* construct was stably introduced into wild-type *Arabidopsis*, and a line showing ∼20% of the native mRNA level was selected for analysis (Figure S1D). Similar to *sp1* plants,^14^ both *spl1* and *SPL2-KD* plants were visibly indistinguishable from wild type under standard growth conditions (Figure S1E).

**Figure 2:**
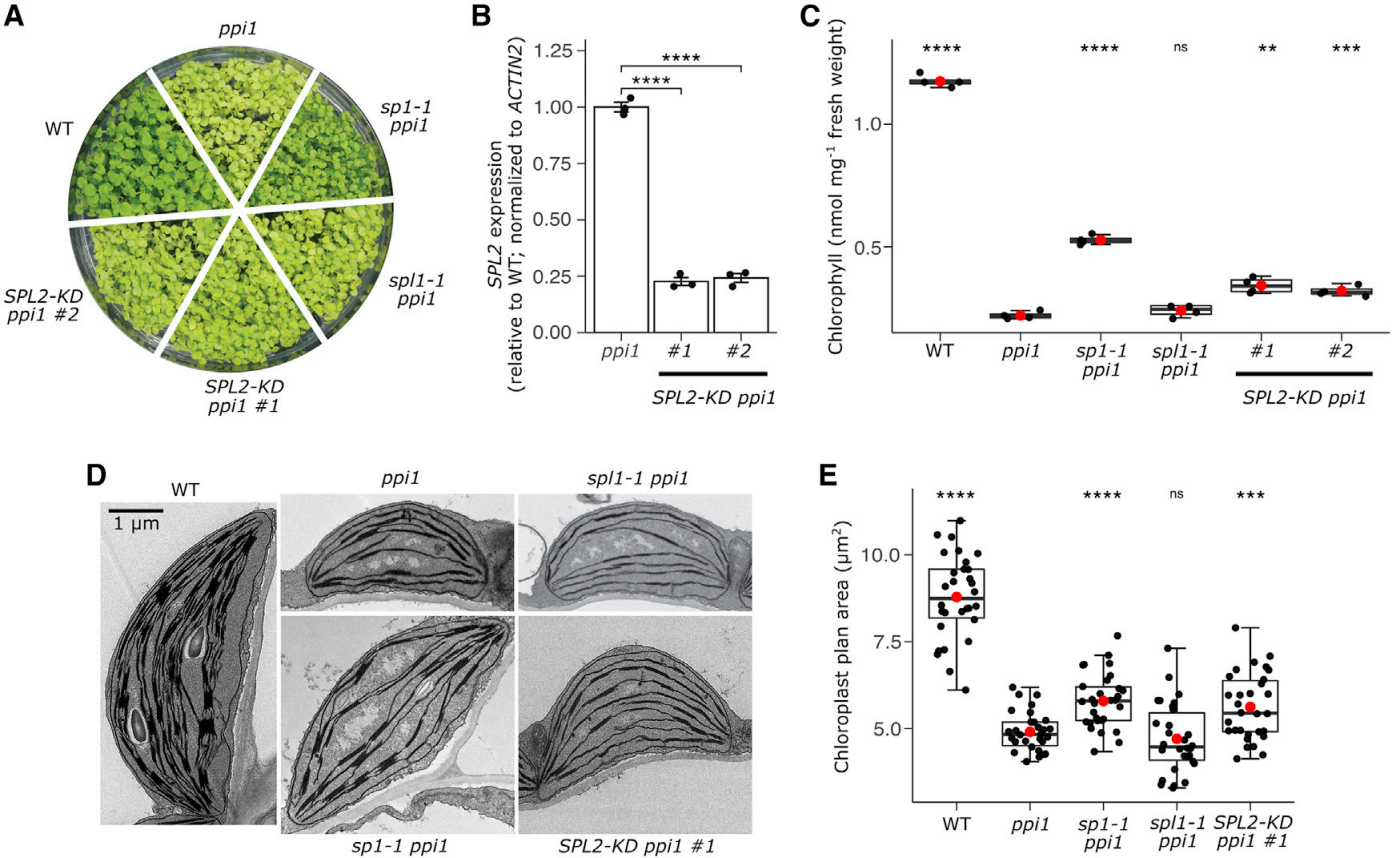
Inactivation of *SPL2*, but not *SPL1*, results in suppression of the *ppi1* phenotype (A) Visible appearance of the different plant genotypes. Wild-type, *ppi1*, *sp1-1 ppi1*, *spl1-1 ppi1*, *SPL2-KD ppi1 #1*, and *SPL2-KD ppi1 #2* plants were grown on MS medium under standard growth conditions for 2 weeks before photography. (B) Assessment of *SPL2* expression in the knockdown lines by qRT-PCR. The analysis was performed using gene-specific primers for *SPL2* and the reference gene *ACTIN2*. The data are shown as mean ± SEM from three independent experiments. (C) Chlorophyll content analysis of the different genotypes. Chlorophyll was measured photometrically following DMF extraction from 10-day-old seedlings grown *in vitro*. Extraction was from 10 seedlings per genotype for each measurement. A total of four independent experiments were performed for each genotype, and the data were presented as boxplots (see below for details). (D and E) Transmission electron microscopy analysis of chloroplast development. Rosette leaf tissue from 35-day-old plants grown on soil under standard growth conditions was fixed and analyzed. Representative micrographs showing typical chloroplasts from each genotype are shown (D). Scale bar indicates 1 μm. Micrographs similar to those in (D) of 30 chloroplasts per genotype were quantitatively analyzed, and the calculated organelle plan area data were presented as boxplots (E). In (C) and (E), the central box represents values between the first and the third quartiles, and the middle line represents the median value. The whiskers extend to the minimum and maximum values. The red circles indicate the means. The black circles in (B), (C), and (E) show the individual data points. For (B), (C), and (E), the p values were derived from Student’s t test analysis (two-tailed) of the means of the indicated genotypes (ns, not significant; ^∗∗^p < 0.01; ^∗∗∗^p < 0.001; ^∗∗∗∗^p < 0.0001). The *ppi1* genotype was used as the reference group for the statistical analysis. See also Figure S1.

The *spl1-1* and *SPL2-KD* lines were each then crossed to *ppi1* to generate the *spl1-1 ppi1* and *SPL2-KD ppi1 #1* double mutants (Figure 2A). In parallel, a second *SPL2-KD* line (termed *SPL2-KD ppi1 #2*) was generated by direct transformation into *ppi1* (Figure 2A), and in both lines, *SPL2* expression was reduced to ∼25% of the native level (Figure 2B).

The *spl1-1 ppi1* double mutants did not display visible or chlorophyll content differences from *ppi1* control plants (Figures 2A and 2C), and this observation was confirmed using a different allele, *spl1-2* (Figures S1F and S1G). In contrast, appreciable recovery of the chlorotic *ppi1* phenotype was apparent in both *SPL2-KD ppi1* lines (Figure 2A), and this was reflected in chlorophyll content measurements (Figure 2C). Moreover, the phenotypic recovery was linked to improved chloroplast development: the chloroplasts in *SPL2-KD ppi1* were larger and more developed than those in *ppi1* (Figures 2D and 2E). In contrast, chloroplasts in *spl1-1 ppi1* were indistinguishable from those in *ppi1* (Figures 2D and 2E).

**Figure 3:**
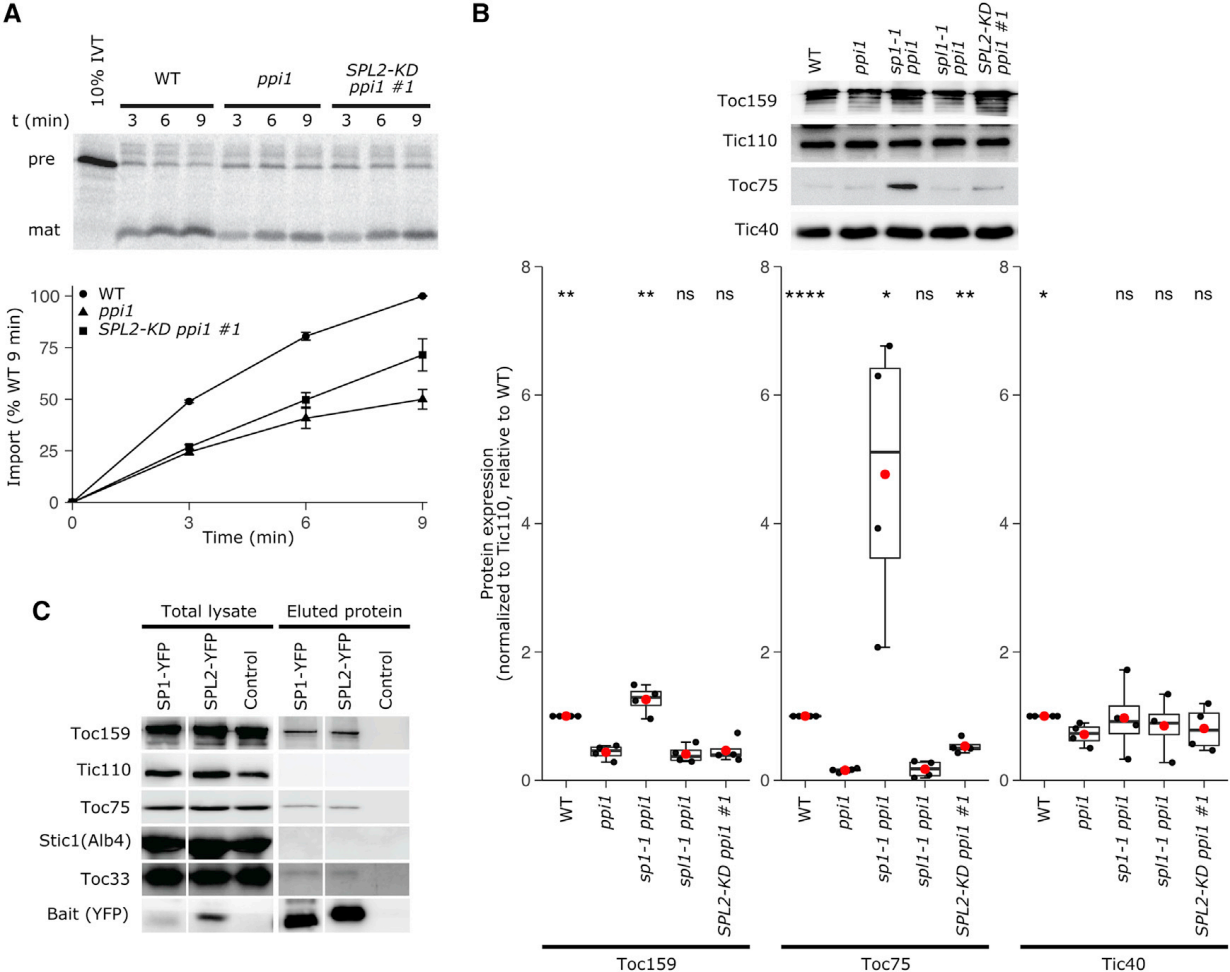
Suppression mediated by *SPL2* knockdown is linked to the chloroplast protein import machinery (A) *In vitro* chloroplast protein import assays. Chloroplasts isolated from 14-day-old wild-type, *ppi1*, and *SPL2-KD ppi1 #1* plants were incubated with *in vitro* translated (IVT) Rubisco small subunit preprotein (pre) and processing to the mature (mat) form was assessed over a time course (3, 6, and 9 min). The amount of mature imported protein at each time point was quantified and expressed as a percentage of the amount of protein imported into wild-type chloroplasts at 9 min. The data shown are means ± SEM from three independent experiments. (B) Immunoblot analysis of TOC protein accumulation. Total protein samples extracted from 2-week-old seedlings of the indicated genotypes were analyzed by immunoblotting using different TOC and TIC antibodies. Bands from three independent experiments were quantified, and the data are shown as boxplots. The central box represents values between the first and third quartiles, and the middle line represents the median value. The whiskers extend to the minimum and maximum values. The red circles indicate the means, and the black circles show the individual data points. The p values were derived from Student’s t test analysis (two-tailed) of the means of the indicated genotypes (ns, not significant; ^∗^p < 0.05; ^∗∗^p < 0.01; ^∗∗∗∗^p < 0.0001). The *ppi1* genotype was used as the reference group for the statistical analysis. (C) Immunoprecipitation analyses of physical interactions with TOC proteins. The SP1 and SPL2 proteins were transiently expressed as YFP fusions in *Arabidopsis* protoplasts, and employed as immunoprecipitation baits. The SP1-YFP fusion was included as a positive control for TOC interaction, whereas a negative control sample lacking a fusion protein was used as a negative control to confirm the specificity of the detected interactions. Protein samples were analyzed by immunoblotting using antibodies against: the YFP tag (anti-GFP was employed), to verify the enrichment of the fusion proteins; TOC components, to assess for substrate interactions; the Tic110 and Stic1/Alb4 proteins, to confirm the specificity of the detected TOC interactions. The low level of SP1-YFP detected in the total lysate sample was as expected, and likely due to the autoregulatory properties of the SP1 protein.^14^

To understand the molecular basis of the chloroplast developmental recovery in *SPL2-KD ppi1*, chloroplast protein import assays were conducted. Chloroplasts isolated from *SPL2-KD ppi1* and control plants were incubated independently with radiolabeled Rubisco small subunit preprotein, and protein uptake was monitored. Chloroplast protein import in *SPL2-KD ppi1* was improved relative to that in *ppi1* (Figure 3A). This recovery in import capacity was linked to stabilization of the TOC apparatus, as *SPL2-KD ppi1* plants displayed elevated abundance of the Toc75 channel (Figure 3B). That the recovery in Toc75 levels in *SPL2-KD ppi1* was quantitatively smaller than that in *sp1 ppi1* plants (which are null for *SP1*) was perhaps because *SPL2* gene silencing was incomplete (Figure 2B). This might also explain the absence of a significant recovery in the abundance of the receptor, Toc159, which even in *sp1 ppi1* showed only modest recovery.^14^

To investigate the possibility that TOC proteins are regulated directly by SPL2 (as they are by SP1), immunoprecipitation (IP) analysis employing YFP-tagged SPL2 (and SP1) was conducted. All the core TOC components (Toc159, Toc75, and Toc33) were detected in fractions that co-eluted with either SPL2-YFP or SP1-YFP but not in control elutions (Figure 3C), and the specificity of the interactions was demonstrated by the absence of non-TOC components (Tic110 and Stic1/Alb4) in the elutions. Thus, we concluded that SPL2 may regulate TOC proteins directly.

### SPL1 lacks E3 ligase activity *in vitro*

Our genetic analyses revealed no suppression of the *ppi1* phenotype in *spl1 ppi1* plants (Figures 2 and 3B). This lack of suppression was not linked to an obvious defect in the E3-defining RING domain of SPL1: RING sequence alignment analysis revealed no obvious differences between SP1 and SPL1 (Figure S2A), and structural models of the cytosolic domains of SP1, SPL1, and SPL2 were similar (Figure S2B). These observations prompted us to investigate whether SPL1 and SPL2 possess E3 ligase activity, similar to SP1, by conducting *in vitro* auto-ubiquitination assays.^14^

**Figure 4:**
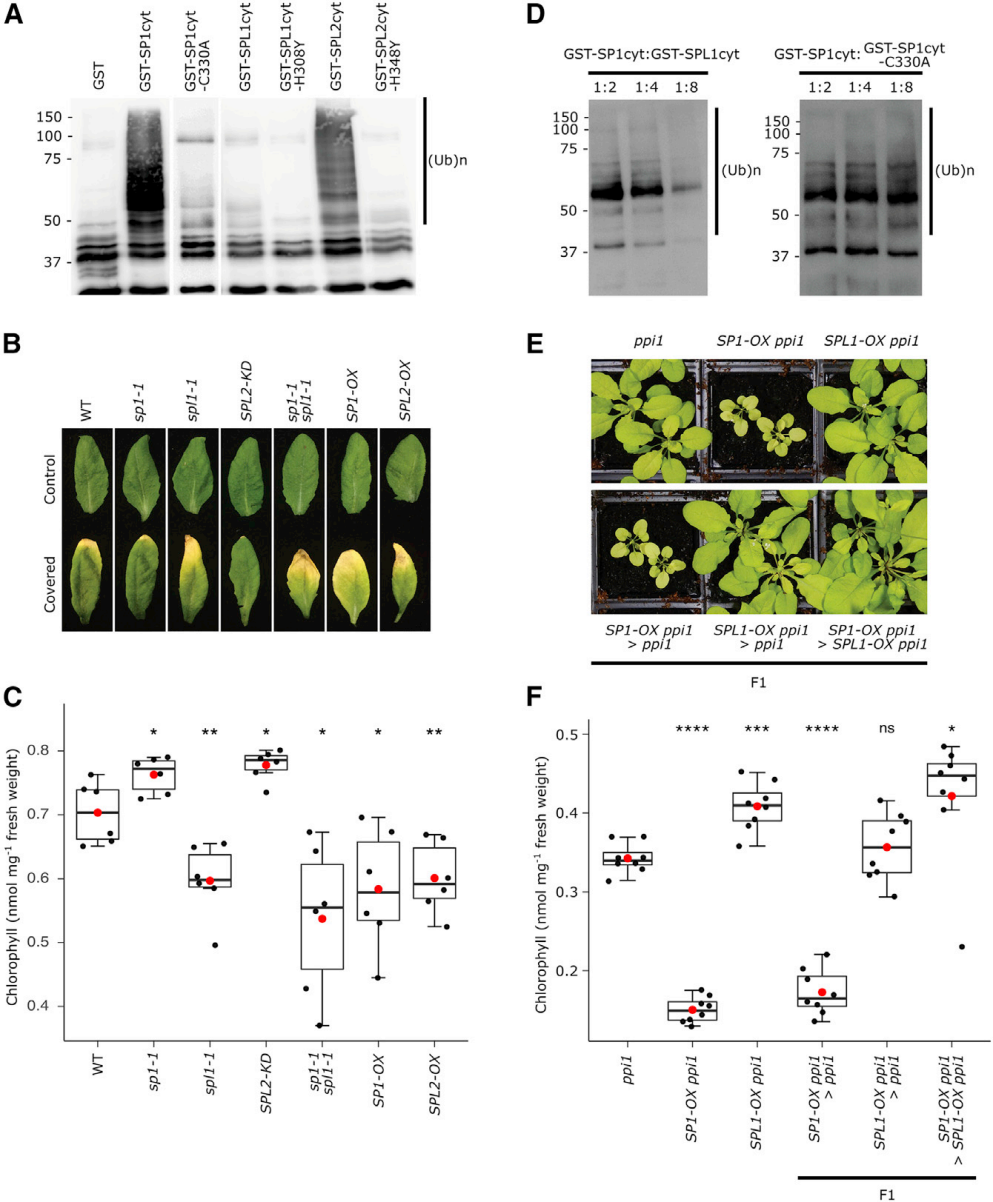
SPL1 lacks E3 ligase activity but acts in opposition to SP1 and SPL2 (A) *In vitro* auto-ubiquitination analysis of SP1 and SPL1. Bacterially expressed GST and GST fusions of the indicated SP1/SPL cytosolic domains (cyt; this domain was previously shown to be the minimal requirement for activity with SP1)^14^ were incubated in a standard reaction containing all other components required for ubiquitination (human E1, 6×His-tagged AtUBC8 E2, and HA-tagged ubiquitin). A mutant version of each protein was included as a negative control; these mutants were generated by substituting a conserved residue in the C3HC4 RING domain.^14^^,^^17^ The reaction products were resolved by immunoblotting using anti-HA antibody, to detect ubiquitinated species. Ubiquitination activity was indicated by the presence of high molecular weight bands of varying sizes, as indicated by the bar labeled (Ub)n. (B and C) Analysis of effects on leaf senescence. Rosette leaves of 28-day-old plants were induced to senesce by covering with aluminum foil for 5 days. The leaves were excised at the end of the dark treatment and photographed (B); control leaves were left uncovered. Chlorophyll contents of the indicated genotypes were measured using a Konica Minolta SPAD-502 meter; data were converted to chlorophyll values as nmol per mg fresh weight (C). The data were derived from six different individuals and are presented as boxplots (see below for details). (D) Analysis of the inhibitory effect of SPL1 on SP1 auto-ubiquitination activity *in vitro*. Bacterially expressed GST-tagged proteins as described in (A) were mixed in different ratios with GST-SP1cyt, as indicated, and then the mixtures were employed in standard ubiquitination reactions as before. Reactions products were analyzed by immunoblotting as in (A). (E and F) Analysis of the effects of combined overexpression of SP1 and SPL1. Visible appearance of plants carrying different combinations of the *SP1-OX* and *SPL1-OX* transgenes in the *ppi1* mutant background. Plants shown in the lower panel are the F1 progeny of the indicated crosses. Plants were grown on soil under standard growth conditions for 28 days before photography (E). Chlorophyll contents of the indicated genotypes were measured using a Konica Minolta SPAD-502 meter; data were converted to chlorophyll values as nmol per mg fresh weight (F). Measurements were recorded from eight different individuals and presented as boxplots. In (C) and (F), the central box represents values between the first and third quartiles, and the middle line represents the median value. The whiskers extend to the minimum and maximum values. The red circles indicate the means, and the black circles show the individual data points. The p values were derived from Student’s t test analysis (two-tailed) of the means of the indicated genotypes (ns, not significant; ^∗^p < 0.05; ^∗∗^p < 0.01; ^∗∗∗^p < 0.001; ^∗∗∗∗^p < 0.0001). The *ppi1* genotype was used as the reference group for the statistical analysis. See also Figures S2–S4.

Polyubiquitin smears (indicating catalytic activity) were observed for both SP1 and SPL2 (Figures 4A and S2C–S2E), indicating that SPL2 is an active E3 ligase. These smears were absent in reactions containing the corresponding RING mutants (C330A and H348Y, respectively), confirming the specificity of the results and the importance of the mutated residues for functionality. However, no ubiquitination activity was detected for SPL1 or its RING mutant (H308Y), using two different E2 enzymes (Figures 4A and S2C–S2E). This implies that SPL1 does not possess similar E3 activity to SP1 and SPL2, which may indicate that SPL1 is a non-functional enzyme; alternatively, SPL1 may require an E2 different from those tested, be subject to autoinhibition that cannot be overcome *in vitro*, have distinct substrate requirements, or be unable to catalyze formation of polyubiquitin chains.

### *SPL1* and *SPL2* are both important during leaf senescence

Plastid transition processes (such as those occurring during leaf senescence) are delayed in *sp1* mutants and promoted in *SP1* overexpressor (*SP1-OX*) plants.^14^ To assess whether the SPL2-SP1 redundancy extends to such transitions, we analyzed the effect of *SPL2* (and, for comparison, *SP1* and *SPL1*) on senescence induction by dark treatment.

Before dark treatment, all genotypes were phenotypically similar to the wild type (Figures S3A and S3B), but upon senescence induction, clear differences became apparent (Figure 4B). Effects of *SPL2* manipulation resembled closely those of *SP1* manipulation: *SPL2-KD* exhibited delayed senescence, whereas *SPL2-OX* exhibited enhanced senescence, and these effects were quantified by measuring chlorophyll content (Figure 4C) and photosynthetic efficiency (Figure S3C). A different, unanticipated effect was observed in *spl1-1*: senescence was accelerated, closely resembling the effect of *SP1-OX* (Figures 4B, 4C, and S3C). This phenomenon was consistent across different *spl1* alleles (Figure S3D). Furthermore, *sp1-1 spl1-1* leaves also showed rapid senescence (Figures 4B, 4C, and S3C), indicating that *spl1* was epistatic over *sp1* in this regard.

In accordance with the notion that *SP1* and *SPL1* exert opposing effects on plant development (an idea also suggested in a previous study, in a different context^20^), whereas the expression of *SP1* increased during senescence, that of *SPL1* decreased markedly in senescent tissues (Figure S3E).^27^ In contrast, *SPL2* expression remained largely unchanged.

### SPL1 negatively regulates the E3 ligase activity of SP1

Based on the results discussed above, we hypothesized that SPL1 inhibits the activity of SP1 via an unknown mechanism. To test this hypothesis, we performed *in vitro* auto-ubiquitination assays similar to those described earlier, except here the E3 proteins were mixed together in different ratios. In agreement with our hypothesis, a reducing trend of ubiquitination was observed when SPL1 was added in excess of SP1 (Figure 4D). A control experiment using mutant SP1 (C330A) in place of SPL1 did not yield a similar effect on SP1 activity (Figure 4D). Although it is possible that SPL1 inhibited activity in this context by titrating E2 enzyme in a way that the SP1 RING mutant could not, this result implied that SPL1 functions as an inhibitor of SP1 activity. In support of this idea, coIP demonstrated physical interaction of SPL1 with SP1 *in vivo* (Figure S4A).

Next, the phenotypic consequences of overexpressing *SP1* and *SPL1* in the *ppi1* background, either in isolation or in combination, were assessed (Figures 4E, 4F, and S4B). The *SP1-OX ppi1* plants were very small and had lower chlorophyll and TOC protein levels than *ppi1*, as expected.^14^ In contrast, *SPL1-OX ppi1* plants were slightly greener than *ppi1* and had similar TOC protein levels. Most significantly, double overexpressors of *SP1* and *SPL1* in the *ppi1* background closely resembled *SPL1-OX ppi1* plants in relation to chlorophyll content and TOC abundance. Thus, *SPL1* overexpression abrogates the effects of *SP1* overexpression, which aligns with the *in vitro* data (Figure 4D) and supports the hypothesis that SPL1 negatively regulates SP1 activity. Because SPL1 physically interacts with TOC proteins (Figure S4C), in addition to SP1 (Figure S4A), it appears that SPL1 and SP1 both interact with TOC complexes, potentially acting in competition with each other.

## Discussion

The requirement for the TOC-TIC machinery to import >90% of chloroplast proteins is well known,^2^^,^^28^ but the regulation of such import was until recently not well understood. The discovery of SP1 and CHLORAD set a new paradigm by establishing post-translational control of the TOC apparatus by the UPS as a crucial regulatory circuit.^14^^,^^16^

### SPL1 is a negative regulator of SP1

The SPL1 and SPL2 proteins were identified as homologs of SP1,^14^ but reliable information on their evolutionary relationships has been lacking.^24^^,^^29^ Here, SP1 and SPL2 were each observed to form a discrete monophyletic group, with SPL1 arising from a duplication event within the SP1 clade (Figure 1A). Elongated SPL1 branch lengths, together with the observation that *SPL1* cannot complement *sp1*,^14^ led us to hypothesize that SPL1 has acquired a function distinct from that of SP1.

Though it possesses an apparently normal RING domain (Figures S2A and S2B), SPL1 lacks E3 activity *in vitro* (Figures 4A and S2E). One possible explanation is that *SPL1* is a pseudogene, as it shares a similar gene structure with its paralogue, *SP1*.^14^^,^^30^ However, this is unlikely because the *SPL1* open reading frame is just five codons shorter than that of *SP1* and *SPL1* expression is robust (Figure S3E). Moreover, clear evidence that SPL1 is functional was provided by the accelerated leaf senescence of *spl1* mutants (Figures 4B, 4C, S3C, and S3D), indicating that SPL1 is important for plastid function and plant development.

The possibility that SPL1 negatively influences SP1 activity was suggested by the opposing *SP1* and *SPL1* expression patterns during leaf senescence (Figure S3E), supported by *in vitro* ubiquitination (Figure 4D) and coIP assays (Figure S4A), and confirmed by analyses of plants overexpressing both *SP1* and *SPL1* (Figures 4E, 4F, and S4B). How SPL1 inhibits SP1 activity is uncertain, but one possibility is that it functions as a dominant-negative protein by competing with SP1 for binding to either substrate proteins or UPS components or by binding to SP1 itself. That SPL1 may act in opposition to SP1 was also suggested by peroxisomal β-oxidation measurements and effects on the physiology and growth of peroxisomal mutants, although E3 activity of SPL1 was not directly assessed.^20^ Most likely, these effects were indirect consequences of SPL1’s inhibitory role in CHLORAD, owing to the close functional links between chloroplasts and peroxisomes.^31^

The emergence of SPL1 as a negative regulator of CHLORAD raised interesting questions concerning the evolutionary events following the founding *SP1/SPL1* duplication event. One possibility is that SPL1 is a functional pseudogenic protein that interferes with the product of the parental gene.^32^^,^^33^ A more likely circumstance is that *SPL1* arose from *SP1* by a process of neofunctionalization.^22^ Why is *SPL1* now restricted to the Brassicaceae, having been lost in other land plant families? The Brassicaceae are recognized as the highest speciation group of land plants,^34^ which may be significant; perhaps the nuanced control of CHLORAD that SPL1 provides is particularly advantageous in these species. It is possible that other species lack this level of control or that proteins other than SPL1 provide regulation. Another possibility is that SP1 in these species has evolved a self-regulation mechanism involving an unidentified SP1 domain; this might explain why the branch lengths outside of the Brassicaceae in the SP1 clade are longer (Figure 1A).

### SPL2 shares incomplete functional redundancy with SP1

Similar to SP1, SPL2 is a chloroplast-localized ubiquitin E3 ligase that selectively targets TOC components to control protein import (Figures 1, 2, and 3). That the *ppi1* suppression delivered by *SPL2-KD* was weaker than that of *sp1* was likely due in part to residual *SPL2* gene expression. However, this also suggests that SP1 is the dominant regulator of the TOC machinery and that the *SP1* gene compensates effectively in the *SPL2-KD* line—a notion consistent with the fact that *SP1*, but not *SPL2*, was identified in the original *ppi1* suppressor screen.^14^

The consequences of *SPL2* expression manipulation for leaf senescence closely mimic those of *SP1* manipulation, revealing an important role for SPL2 in plastid operation and plant development (Figures 4B, 4C, and S3C). In fact, a recent study showed that this role also involves regulation of chromoplast formation during tomato fruit ripening.^35^ It is noteworthy that both SP1 and SPL2 have been implicated in abiotic stress responses: SP1 promotes tolerance of salt, osmotic, and oxidative stresses by reducing chloroplast protein import to limit photosynthesis and overaccumulation of damaging reactive oxygen species,^15^ whereas *SPL2/NERF* was proposed to promote drought tolerance, albeit via a different mechanism.^17^ Differential regulation by two different E3 ligases may enable greater control in response to diverse abiotic stresses.

Regulation of substrates by multiple E3 ligases has been well documented in other systems.^36^^,^^37^ With regard to TOC regulation, whether SP1 and SPL2 function independently or cooperatively is uncertain. What is clear, though, is that the functions of SP1 and SPL2 do not completely overlap. Thus, SPL2 may act on additional chloroplast substrates that are not regulated by SP1 (which is strongly focused on TOC regulation). The existence of multiple chloroplast E3 ligases with distinct substrate ranges supports the view that the UPS has an even greater role in chloroplast biology than was previously envisaged and that the scope of CHLORAD is not limited to protein import control.^38^

## STAR★Methods

### Key resources table

**Table:** 

REAGENT or RESOURCE	SOURCE	IDENTIFIER
**Antibodies**		

anti-atStic1/Alb4	Trösch et al ^39^	N/A
anti-atTic110	Aronsson et al^40^	N/A
anti-atTic40	Kasmati et al ^41^	N/A
anti-atToc159	Bauer et al ^42^	N/A
anti-atToc33	Aronsson et al ^43^	N/A
anti-atToc75-III	Kasmati et al ^41^	N/A
anti-H3	Abcam	Cat#ab1791; RRID: AB_302613
anti-GFP	Sigma	Cat#SAB4301138; RRID: AB_2750576
anti-HA tag	Sigma	Cat#H6908; RRID: AB_260070
anti-Myc	Abcam	Cat#ab9106; RRID: AB_307014
anti-rabbit IgG HRP	Sigma	Cat#12-348; RRID: AB_390191

**Bacterial and virus strains**		

α-Select Silver Competent Cells	Bioline	Cat#BIO-85026
*Agrobacterium tumefaciens* GV3101 (pMP90)	Karimi et al ^44^	N/A
*Escherichia coli* Rosetta (DE3) cells	Sigma	Cat#70954

**Chemicals, peptides, and recombinant proteins**		

precursor of small subunit of Rubisco (pSSU)	Aronsson and Jarvis^45^	N/A
^35^S-methionine	Perkin-Elmer	Cat#NEG072002MC
6×His-tagged AtUBC8 E2	Ling et al^14^	N/A
human UbcH5b	Boston Biochem	Cat#E2-622
bortezomib	Selleckchem	Cat#S1013
Protease Inhibitor Cocktail	Sigma	Cat#P9599
DUCHEFA Cellulase R-10	Melford Biolaboratories Limited	Cat#C8001.0010
EZ-Chemiluminescence Detection Kit for HRP	Geneflow	Cat#K1-0170
GFP-Trap MA magnetic beads	Chromotek	Cat#gtma-20
EZview Red Anti-c-Myc Affinity Gel	Sigma	Cat#E6654
EZview Red Protein A Affinity Gel	Sigma	Cat#P6486
c-Myc Peptide	Merck	Cat#M2435
GST	Ling et al^14^	N/A
GST-SP1cyt	Ling et al^14^	N/A
GST-SP1cyt-C330A	Ling et al^14^	N/A
GST-SPL1cyt	This paper	N/A
GST-SPL1cyt-H308Y	This paper	N/A
GST-SPL2cyt	This paper	N/A
GST-SPL2cyt-H348Y	This paper	N/A
HA-ubiquitin	R&D systems-Boston Biochem	Cat#U-110-01M
human E1	Sigma	Cat#U5633
macerozyme R-10	Melford Biolaboratories Limited	Cat#M8002.0005
Percoll	Sigma	Cat#GE17-0891-01
qPCRBIO SyGreen Mix Hi-ROX	PCR Biosystems	Cat#PB20.12-20
SP1-YFP	Ling et al^14^	N/A
SP1-Myc	Ling et al^16^	N/A
SPL1-YFP	Ling et al^14^	N/A
SPL1-HA	This paper	N/A
SPL2-YFP	Ling et al^14^	N/A
YFP-HA	Ling et al^14^	N/A
SuperScript IV Reverse Transcriptase	Invitrogen	Cat#18090200
TNT T7 Quick for PCR DNA	Promega	Cat#L5540

**Experimental models: Organisms/strains**		

*Arabidopsis*: *ppi1*: *ppi1-1*	Jarvis et al^23^	N/A
*Arabidopsis*: *sp1*: *sp1-1*	Ling et al^14^	N/A
*Arabidopsis*: *spl1-1*: *spl1-1*	Arabidopsis Biological Resource Center	salk_064720
*Arabidopsis*: *spl1-2*: *spl1-2* (*dal2-1*)	Arabidopsis Biological Resource Center	salk_024744
*Arabidopsis*: *spl1-3*: *spl1-3* (*dal2-2*)	Arabidopsis Biological Resource Center	WiscDsLox383C6
*Arabidopsis*: *SP1-OX*: *SP1-OX*	Ling et al^14^	N/A
*Arabidopsis*: *sp1-1 ppi1*: *sp1-1 ppi1-1*	Ling et al^14^	N/A
*Arabidopsis*: *sp1-3 ppi1*: *sp1-3 ppi1-1*	Ling et al^14^	N/A
*Arabidopsis*: *SP1-OX ppi1*: *SP1-OX ppi1-1*	Ling et al^14^	N/A
*Arabidopsis*: *SPL1-OX ppi1*: *SPL1-OX ppi1-1*	Ling et al^14^	N/A
*Arabidopsis*: *SPL2-OX sp1-3 ppi1*: *SPL2-OX sp1-3 ppi1-1*	This paper	N/A
*Arabidopsis*: *SPL2-KD ppi1 #1*: *SPL2-KD ppi1-1 #1*	This paper	N/A
*Arabidopsis*: *SPL2-KD ppi1 #2*: *SPL2-KD ppi1-1 #2*	This paper	N/A
*Arabidopsis*: *SPL2-KD*: *SPL2-KD*	This paper	N/A
*Arabidopsis*: *sp1-1 spl1-1*: *sp1-1 spl1-1*	This paper	N/A
*Arabidopsis*: *SPL2-OX*: *SPL2-OX*	This paper	N/A
*Arabidopsis*: *spl1-2 ppi1*: *spl1-2 ppi1-1*	This paper	N/A
*Arabidopsis*: *SPL1-OX ppi1*: *SPL1-OX ppi1-1*	This paper	N/A
*Arabidopsis*: *SP1-OX ppi1* > *ppi1* F1: *SP1-OX ppi1-1 > ppi1-1* F1	This paper	N/A
*Arabidopsis*: *SPL1-OX ppi1* > *ppi1* F1: *SPL1-OX ppi1-1 > ppi1-1* F1	This paper	N/A
*Arabidopsis*: *SP1-OX ppi1* > *SPL1-OX ppi1* F1: *SP1-OX ppi1-1 > SPL1-OX ppi1-1* F1	This paper	N/A

**Oligonucleotides**		

See Table S1 for oligonucleotides	N/A	N/A

**Software and algorithms**		

Aida Image Analyzer	Raytest	http://www.elysia-raytest.com
MergeAlign	Collingridge and Kelly^46^; Katoh et al^47^	https://mergealign.appspot.com
IQ-TREE	Trifinopoulos et al^48^	http://iqtree.cibiv.univie.ac.at
iTOL	Letunic and Bork ^49^	https://itol.embl.de
I-TASSER	Zhang ^50^; Roy et al ^51^	https://zhanglab.ccmb.med.umich.edu/I-TASSER/
PyMOL	Schrödinger	https://pymol.org/2/
Fiji (ImageJ)	Schindelin et al ^52^	https://imagej.nih.gov/ij/
RStudio	The R Foundation	https://www.r-project.org
Web MicroRNA Designer	Ossowski et al ^25^; Schwab et al ^26^	http://wmd3.weigelworld.org/cgi-bin/webapp.cgi

### Resource availability

#### Lead contact

Further information and requests for resources and reagents should be directed to and will be answered by the Lead Contact, R. Paul Jarvis (paul.jarvis@biology.ox.ac.uk).

#### Materials availability

Plasmids and plant lines generated in this study can be obtained through the Lead Contact.

### Experimental model and subject details

All *Arabidopsis thaliana* plants used in this study were of the Columbia-0 (Col-0) ecotype. Transgenic plant lines were generated by agrobacterium-mediated transformation. Seeds were either germinated on petri dishes containing Murashige and Skoog (MS) medium (4.3 g L^-1^ MS basal salt mixture, 0.5% [w/v] sucrose, 0.05% [w/v] 2-[*N*-morpholino]ethanesulfonic acid [MES], pH adjusted to 5.7 with potassium hydroxide [KOH]; phyto-agar was added to a concentration of 0.65% [w/v] before sterilization by autoclaving at 110°C for 15 minutes), or on soil (Levington Advance Seed and Modular F2). Where necessary, 10 μg mL^-1^ DL-phosphinothricin (Duchefa) was included in the MS medium for the selection of transformants. For in vitro growth, seeds were surface sterilized for 5 minutes with 70% (v/v) ethanol and 0.05% (v/v) Triton X-100, followed by an additional 10 minutes with 100% (v/v) ethanol before sowing. Plates were stratified in the dark at 4°C for a minimum of 2 days. Plants grown in vitro or on soil were kept under standard growth conditions at 22-24°C with a long-day photoperiod (16 hours light, 8 hours dark) of 120 μmol m^-2^ s^-1^ white light.

### Method details

#### Physiological analysis

Chlorophyll content analysis of *Arabidopsis* seedlings was performed using a spectrophotometer following extraction with *N*,*N*’-dimethylformamide (DMF), as previously described.^53^ For mature plants, chlorophyll levels were measured using a Konica-Minolta SPAD-502 meter with the recorded values converted into nmol per mg fresh weight using a published equation.^54^

Induction of senescence by dark treatment was performed on equally-developed leaves of 28-day-old plants using previously described procedures.^14^ Photosynthetic performance (maximum photochemical efficiency of photosystem II, *F*_*v*_/*F*_*m*_) was recorded using a CF Imager chlorophyll fluorescence imaging system (Technologica, Essex, UK) as described previously.^15^

#### Generation of transgenic plant lines

To generate the *SPL2* cassette for the generation of *SPL2* overexpression (OX) lines, the full-length *Arabidopsis SPL2* coding sequence (CDS) was amplified by PCR from wild-type cDNA using the following primers: SPL2-cDNA-F, 5’-AAAAAGCAGGCTCCACTTGTCCGTGTGACCG-3’; and SPL2-cDNA-R, 5’-AGAAAGCTGGGTTATCGGTTACAAAATTTCTTCC-3’. All primers used in this study are listed in Table S1.

To prepare the artificial microRNA (amiRNA) cassette for the generation of *SPL2* knockdown (KD) lines, the original miR319a sequence in the pRS300 plasmid was replaced with an *SPL2* target sequence, essentially as described previously.^26^ The following primers were designed using the Web MicroRNA Designer (WMD) platform^25^ and used: SPL2amR1-I miR-s, 5’- GATACTAAGAGTAATATACGCGCTCTCTCTTTTGTATTCC-3’; SPL2amR1-II miR-a, 5’-GAGCGCGTATATTACTCTTAGTATCAAAGAGAATCAATGA-3’; SPL2amR1-III miR^∗^s, 5’-GAGCACGTATATTACACTTAGTTTCACAGGTCGTGATATG-3’; and SPL2amR1-IV miR^∗^a, 5’- GAAACTAAGTGTAATATACGTGCTCTACATATATATTCCT-3’.

The *SPL2* CDS and the *SPL2* amiRNA cassettes were each cloned into the cauliflower mosaic virus (CaMV) 35*S*-promoter-driven binary overexpression vector pB2GW7^44^ using the Gateway cloning system (Invitrogen), generating the *SPL2-OX* and *SPL2-KD* constructs, respectively. The constructs were introduced into *Arabidopsis* wild-type, *sp1-3 ppi1*, or *ppi1* plants using the floral dip method.^55^ At least 10 independent T1 lines were analysed per construct, and single-locus T2 lines showing a 3:1 segregation of resistance on selective medium were propagated for further analysis.

#### Characterization of mutant and transgenic plant lines

Analysis of gene expression by semi-quantitative or quantitative RT-PCR, analysis of protein levels by immunoblotting, and chloroplast isolation and protein import assays were all performed as described previously.^15^^,^^45^^,^^56^^,^^57^

The primers used for semi-quantitative RT-PCR were: *SPL2*, 5’-AAAAAGCAGGCTCCATGTCCTCGCCGGAGCGTG-3’ and 5’- AGAAAGCTGGGTTCTAAGAGTAATATACACGCATAGAT-3’; and *eIF4e1*, 5’- AAACAATGGCGGTAGAAGACACTC-3’ and 5’-AAGATTTGAGAGGTTTCAAGCGGTGTAAG-3’. The primers used for quantitative RT-PCR were: *SPL2*, 5’-ATGACCAAGGACAAGATGA-3’ and 5’- ATGCCAACAGACACAATG-3’; and *ACTIN2*, 5’-TCAGATGCCCAGAAGTCTTGTTCC-3’ and 5’-CCGTACAGATCCTTCCTGATATCC-3’.

Immunoblotting was performed using a standard procedure as described previously.^15^^,^^57^ Primary antibodies used to detect the chloroplast proteins were as follows: anti-atToc159,^42^ anti-atToc75-III,^41^ anti-atToc33,^43^ anti-atTic110,^40^ anti-atTic40,^41^ and anti-atStic1/Alb4.^39^ Other primary antibodies employed in this study were: anti-Histone H3 (Abcam), anti-GFP (green fluorescent protein) (Sigma), anti-Myc (Abcam), and anti-HA (haemagglutinin) tag (Sigma). Anti-rabbit immunoglobulin G (IgG) conjugated with horseradish peroxidase (Abcam) was employed as the secondary antibody. Bands were detected using an EZ-Chemiluminescence Detection Kit for HRP (Geneflow), and recorded using an LAS-4000 imager (GE Healthcare). Band intensities were quantified using Aida Image Analyzer software (Raytest).

#### Immunoprecipitation

Isolation of protoplasts from the rosette leaves of 4-week-old wild-type plants, and their use for the transient expression of tagged proteins, was conducted as described previously.^15^^,^^58^ The *SPL1-HA* construct was generated by subcloning the *SPL1* coding sequence (CDS)^14^ into a modified p2GW7 plant expression vector providing a C-terminal hemagglutinin (HA) tag; and the *SPL2-YFP* construct used was generated by cloning the *SPL2* CDS (amplified using SPL2-CDS-F, 5’- AAAAAGCAGGCTCCATGTCCTCGCCGGAGCGTG-3’; and SPL2-nonstop-R, 5’- AGAAAGCTGGGTTAGAGTAATATACACGCATAG-3’) into the pK7YWG2 binary vector.^44^ The *SP1-YFP*, *SP1-Myc*, *SPL1-YFP* and *YFP-HA* constructs have been described previously.^14^ The proteasome inhibitor bortezomib (Selleckchem) was added to the protoplast culture medium after 15 hours of incubation following transfection, to a final concentration of 5 μM; cultures were then incubated for an additional 4 hours before analysis.

Immunoprecipitation of the YFP fusion proteins was carried out using a previously described procedure,^14^^,^^16^ with minor modifications. Following cell lysis, total protein extracts were incubated with 25 μL GFP-Trap MA magnetic beads (Chromotek) for 1 hour on a rotating mixer at 4°C. A MagJET separation rack (Thermo Fisher) was used to pellet the beads and any bound protein complexes. After four rounds of washing, each one with 500 μL wash buffer (25 mM Tris-HCl, pH 7.5, 150 mM NaCl, 10% [v/v] glycerol, 1 mM EDTA, 0.5% [v/v] Triton-X100), the bound proteins were eluted by boiling in 2× protein loading buffer (60 mM Tris-HCl, pH 6.8, 10% [v/v] glycerol, 2% [w/v] sodium dodecyl sulphate [SDS], 0.05% [w/v] bromophenol blue, 0.1 M dithiothreitol [DTT]) for 5 minutes, and analysed by SDS-PAGE and immunoblotting.

#### Phylogenetic analysis

The amino acid sequences of the SP1, SPL1 and SPL2 homologues employed in the analysis were retrieved by using the *Arabidopsis thaliana* sequences as queries to perform BLASTP searches of the Phytozome,^59^ Ensembl Plants,^60^ and National Center for Biotechnology Information (NCBI) databases. All identified plant accessions with a complete RING domain sequence were retrieved and checked by performing reciprocal BLAST searches against the *A. thaliana* genome. Plant species that were represented by sequences truncated in the highly-conserved C3HC4 RING domain were not included in the analysis. The related human mitochondrial outer-membrane protein MULAN/MAPL (NM_024544) was included in the analysis as an outgroup. The sequences were aligned using MergeAlign,^46^^,^^47^ and phylogeny was inferred by maximum likelihood using the IQ-TREE^48^ web server. The interactive Tree Of Life (iTOL) version 4 program^49^ was used to visualise the tree.

#### Protein structural modelling

The protein structure models of the *A. thaliana* SP1 (residues 241-343), SPL1 (residues 240-338), and SPL2 (residues 288-383) cytosolic RING domains were predicted by submitting the corresponding amino acid sequences to the I-TASSER (Iterative Threading Assembly Refinement) online server.^50^^,^^51^ Predicted models with the highest confidence scores (*C*-scores) were selected and visualized using PyMOL (https://pymol.org/2/).

#### Transmission electron microscopy

Transmission electron microscopy (TEM) was performed on 4-week-old developmentally equivalent leaves as described previously,^61^ with several modifications as follows: 2 mm discs were excised from leaves using a biopsy punch, then cut in half with a scalpel. Leaf samples were cut in a droplet of primary fixative (2.5% [v/v] glutaraldehyde and 4% [v/v] paraformaldehyde [PFA] in 0.1 M sodium cacodylate buffer [SCB], pH 6.9) and fixed in a Leica AMW microwave processing unit at 37°C with five alternating 2-minute cycles of 20 W and 0 W power. The samples were then allowed to fix further at room temperature for ∼5 hours in glass vials over-filled with primary fixative, and then left at 4°C overnight. Prior to secondary fixation, the overnight samples were washed four times, as follows: twice for 45 minutes and 30 minutes in 0.1 M SCB, pH 6.9; once for 30 minutes in 0.1 M SCB, pH 6.9, containing 50 mM glycine; and once for 20 minutes in 0.1 M SCB, pH 6.9. The samples were then incubated in secondary fixative (1% [v/v] osmium tetroxide in 0.1 M SCB, pH 6.9, containing 1.5% [w/v] potassium ferrocyanide) for 2 hours at 4°C, and for a further 50 minutes at room temperature, with rotation. The dehydration series was performed with ice-cold ethanol at 4°C with rotation, and following the final incubation in 100% ethanol the samples were transferred to ice-cold 100% acetone and incubated for 30 minutes at room temperature. Samples were infiltrated with epoxy resin as described,^61^ except that TAAB Hard Plus resin and acetone were used, and the samples were left an extra 24 hours in 100% resin before polymerization.

Chloroplast images were captured at the same magnification level using a Tecnai 12 TEM operated at 120 kV. Three samples per genotype were analysed, and at least ten different whole-chloroplast images per plant sample were recorded (a total of 30 chloroplasts per genotype). Chloroplast cross-sectional area was estimated using Fiji, a distribution of the open-source software package ImageJ.^52^

#### Recombinant proteins and in vitro ubiquitination assays

The expression and purification of GST, GST-SP1cyt (residues 244-343), GST-SP1cyt-C330A (Cys330-to-Ala), GST-SPL1cyt (residues 241-338), GST-SPL1cyt-H308Y (His308-to-Tyr), GST-SPL2cyt (residues 289-383), and GST-SPL2cyt-H348Y (His348-to-Tyr) followed a procedure that has been described previously.^14^ The primers used for the generation of the *SPL*-related constructs were: *SPL1cyt*, 5’-GGGGACAAGTTTGTACAAAAAAGCAGGCTCCGTGATTGAATATATTCTA-3’ and 5’-GGGGACCACTTTGTACAAGAAAGCTGGGTTTCAATGGCGGTAAATTTTC-3’; and *SPL2cyt*, 5’-GGGGACAAGTTTGTACAAAAAAGCAGGCTCCGCTGCTGTCAGGACCTGGAA-3’ and 5’-AGAAAGCTGGGTTCTAAGAGTAATATACACGCATAGAT-3’. The RING mutant *SPL* constructs were generated by combined overlap-extension PCR in vitro^62^ using these additional primers: *SPL1-H308Y*, 5’-GAGTGTGGTTATATGTGCTG-3’ and 5’-CAGCACATATAACCACACTC-3’; and *SPL2-H348Y*, 5’-CCTGCGTTTATTCCCTGTGGATATGTAGTATG-3’ and 5’-CACATCGCCTGCAACATACTACATATCCACAG-3’. The *GST*, *GST-SP1cyt* and *GST-SP1cyt-C330A* constructs have all been described before.^14^

Ubiquitination assays employed a method described previously.^14^ An alternative E2 enzyme, human UbcH5b (Boston Biochem), was also employed in place of 6xHis-tagged AtUBC8 in some assays. For the ubiquitination experiments containing two recombinant E3 ligases, the proteins were mixed in different amount ratios (1:2, 1:4 and 1:8) in the same 30 μL reaction.

### Quantification and statistical analysis

Statistical analysis was carried out using RStudio Version 1.2.5019. The numbers of samples analysed, and the reference group used, are specified in the figure legends. The p values were derived from Student’s t-test analysis (two-tailed) of the means of the indicated genotypes (ns, not significant at p > 0.05; ^∗^, significant at p < 0.05; ^∗∗^, significant at p < 0.01; ^∗∗∗^, significant at p < 0.001, ^∗∗∗∗^, significant at p < 0.0001)

## Data Availability

This study did not generate any unique datasets or code.

## Supplemental information

Document S1. Figures S1–S4 and Table S1

Document S2. Article plus supplemental information
