# Supplementary material for: Multiple ubiquitin E3 ligase genes antagonistically regulate chloroplast-associated protein degradation
Source: Curr Biol. 2023 Mar 27;33(6):1138–1146.e5. doi: 10.1016/j.cub.2023.01.060 (PMC11913770; doi:10.1016/j.cub.2023.01.060)
Supplement: Document S1. Figures S1–S4 and Table S1 [file mmc1.pdf]

Current Biology, Volume *33*

Supplemental Information

Multiple ubiquitin E3 ligase genes antagonistically regulate chloroplast-associated protein degradation

Sabri Mohd. Ali, Na Li, Ziad Soufi, Jinrong Yao, Errin Johnson, Qihua Ling, and R. Paul Jarvis

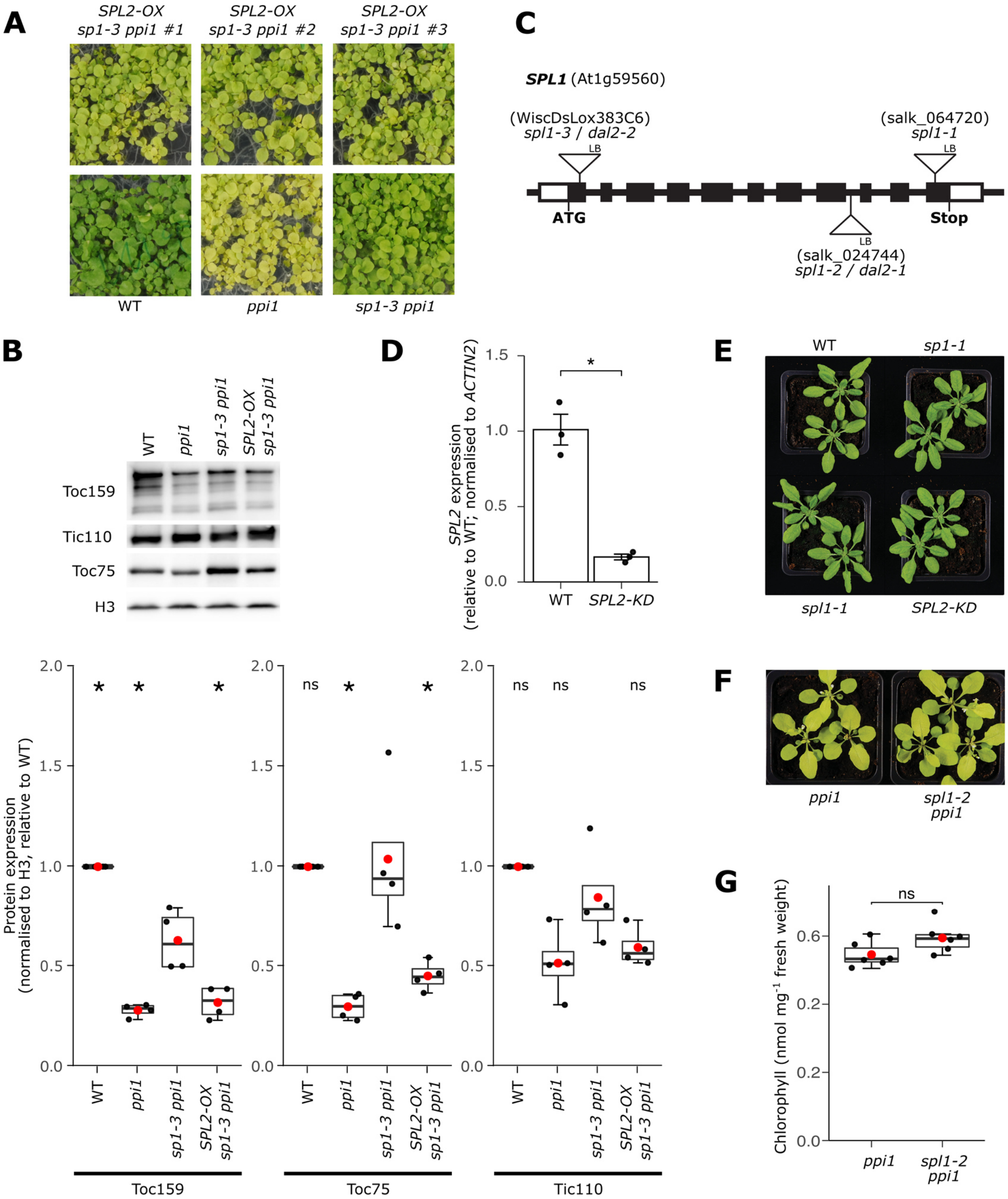

Figure S1. Basic analyses of the *SPL1* and *SPL2* plant lines, related to Figures 1 and 2.

(A) Visible appearance of additional *SPL2* overexpression (OX) lines in the *sp1 ppi1* double mutant background. Plants were grown on MS medium under standard growth conditions for 21 days before photography.

(B) Immunoblot analysis of TOC protein accumulation in *SPL2-OX* plants. Total protein samples extracted from two-week-old seedlings of the indicated genotypes were analysed by immunoblotting using a range of different TOC and TIC antibodies. Bands from four independent experiments were quantified and the data are shown as boxplots. The central box represents values between the first and the third quartiles, and the middle line represents the median value. The whiskers extend to the minimum and maximum values. The red circles indicate the means, and the black circles show the individual data points. The p values were derived from Student's *t*-test (two-tailed) of the means of the indicated genotypes (ns, not significant; *, significant at $p < 0.05$). The *sp1-3 ppi1* genotype was used

as the reference group for the statistical analysis.
(C) Schematic representation of the *SPL1* genomic locus (At1g59560) indicating the position and orientation of each T-DNA insertion. Protein-coding exons = black boxes; untranslated regions = white boxes; introns = black lines between the boxes; LB = T-DNA left border sequence; ATG = translation initiation codon; Stop = translation termination codon.
(D) Assessment of gene expression in the *SPL2* amiRNA knockdown (KD) line by quantitative RT-PCR using gene-specific primers for *SPL2* and the reference gene *ACTIN2*. The data are shown as mean ± SEM from three independent experiments. Black circles show the individual data points. The p value was derived from a Student's *t*-test analysis (two-tailed) of the means (*, significant at $p < 0.05$). The wild type was used as the reference group for the statistical analysis.
(E) Visible appearance of wild-type, *sp1-1, spl1-1* and *SPL2-KD* plants. Plants were grown on soil for 28 days under standard growth conditions before photography. The three *spl1* alleles were phenotypically identical, and so only *spl1-1* is shown as a representative allele.
(F) Visible appearance of *ppi1* and *spl1-2 ppi1* double-mutant plants. Plants were grown on soil under standard growth conditions for 28 days before photography.
(G) Chlorophyll contents of the indicated genotypes were measured using a Konica-Minolta SPAD-502 meter. Meter values were converted to chlorophyll values as nmol per mg fresh weight. Measurements were made from six different individuals and presented as boxplots, as in panel (B). The p value was derived from a Student's *t*-test analysis (two-tailed) of the means (ns, not significant). The *ppi1* genotype was used as the reference group for the statistical analysis.

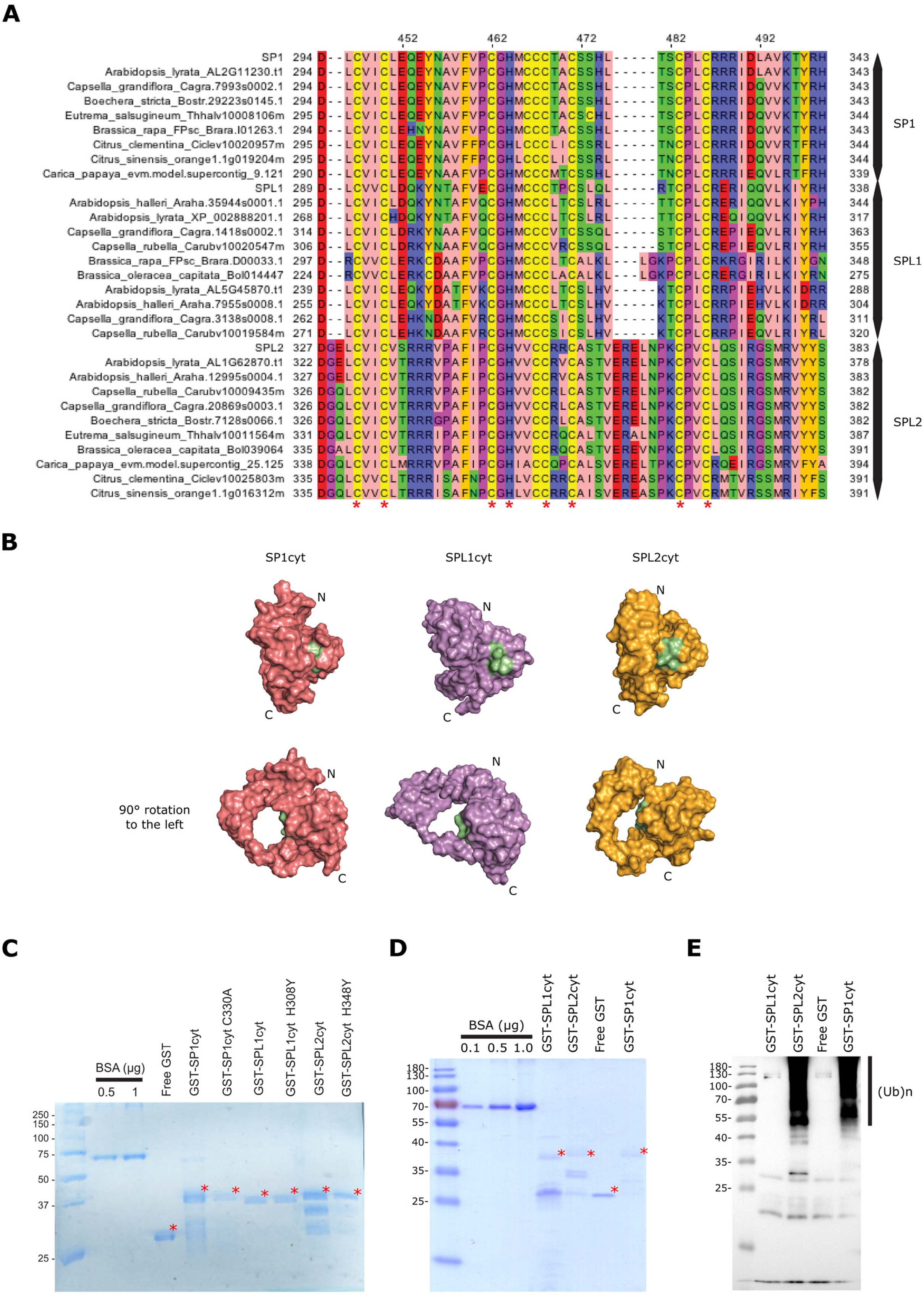

Figure S2. Analyses of the E3 properties of the SP1 and SPL proteins, related to Figure 4.

(A) Multiple sequence alignment of the RING domains of SP1, SPL1 and SPL2 from selected plant species. *A. thaliana* sequences are labelled to the left as SP1, SPL1 and

SPL2. The critical conserved cysteine and histidine residues of the RING finger are indicated with asterisks.
(B) Predicted structures of the *A. thaliana* SP1, SPL1 and SPL2 cytosolic domains. The relevant amino acid sequences were submitted to the web server I-TASSER, and the 3D models thus obtained were visualized using PyMOL. The green region indicates the conserved residues of the RING finger in each case.
(C and D) Analysis of the protein samples used in the autoubiquitination analyses. Bacterially-expressed and purified GST and GST fusions of the indicated SP1/SPL cytosolic domains were resolved by SDS-PAGE and Coomassie staining to asses purity and concentration. Positions of molecular weight standards are indicated at left (sizes are in kDa). The expected positions of the free GST and GST fusion proteins are indicated with red asterisks. Concentrations of the correct-sized purified protein bands were estimated by comparison with known amounts of bovine serum albumin standards. The samples were used to conduct the in vitro autoubiquitination assays in Figure 4 (C) and below in this figure (D).
(E) In vitro autoubiquitination analysis of SP1 and SPL proteins using an alternative E2 conjugase. The protein samples from (D) were incubated in a standard reaction containing all other components required for ubiquitination (human E1, human UbcH5b E2, and HA-tagged ubiquitin). The reaction products were resolved by immunoblotting using anti-HA antibody, to detect ubiquitinated species. Ubiquitination activity was indicated by the presence of high molecular weight bands of varying size, as indicated by the bar labelled (Ub)n. Positions of molecular weight standards are indicated at left (sizes are in kDa).

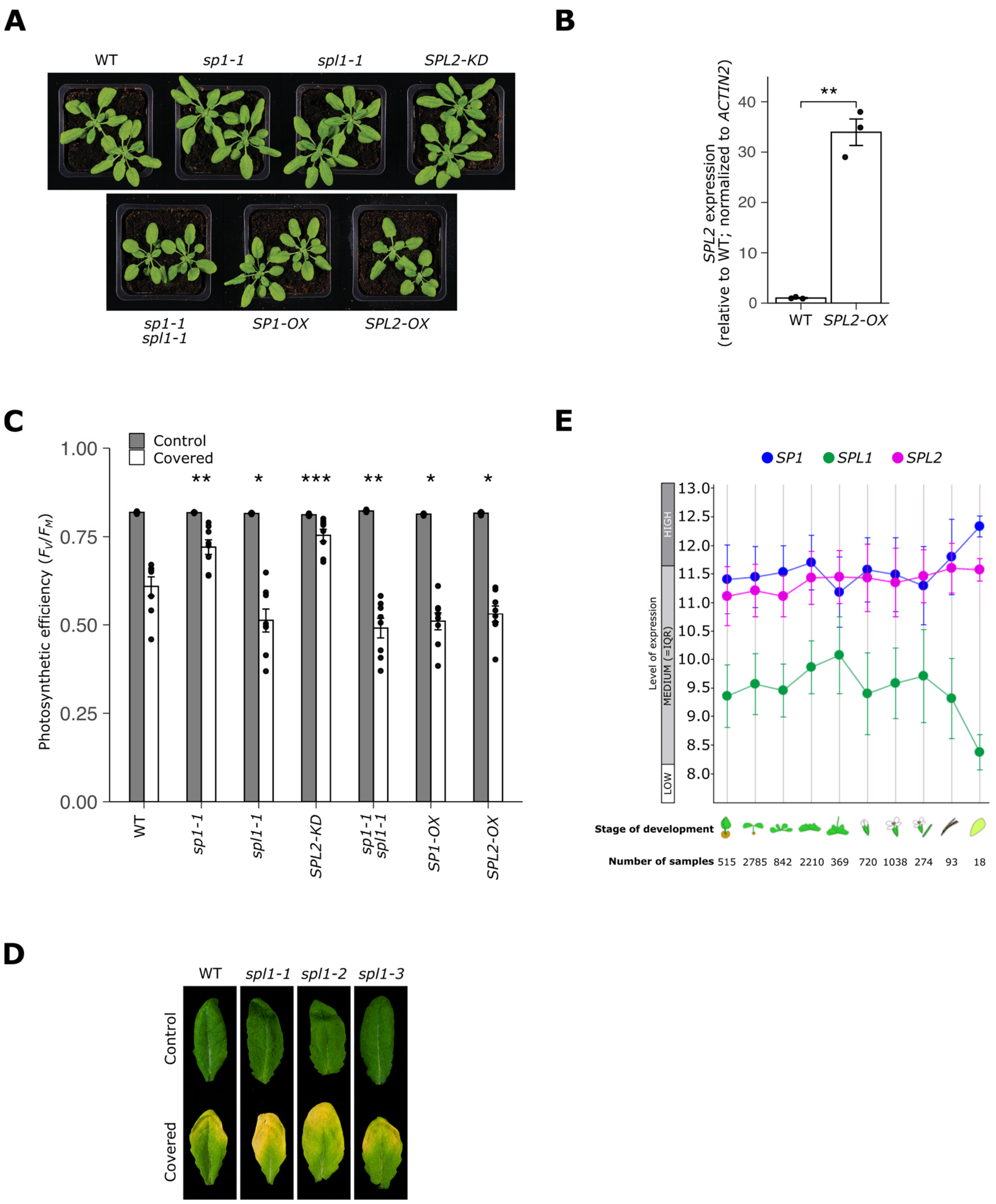

Figure S3. Analyses of the *SP1*/*SPL* plant lines concerning leaf senescence and gene expression data, related to Figure 4.

(A and B) Analyses of the plants prior to the induction of senescence. The plants were grown on soil under standard growth conditions for 21 days before photography, and the different genotypes were visibly indistinguishable (A). Gene expression in the *SPL2* overexpressor plants was assessed by quantitative RT-PCR using gene-specific primers for *SPL2* and the reference gene *ACTIN2* (B). The data are shown as mean ± SEM from three independent experiments. Black circles show the individual data points. The p values were derived from a Student's *t*-test analysis (two-tailed) of the means of the indicated genotypes (**, significant at $p < 0.01$). The wild type was used as the reference group for the statistical analysis.

(C) Analysis of photosynthetic performance following the induction of senescence. The

maximum photochemical efficiency of photosystem II (F_v/F_m) of the plants described in Figure 4 (B and C) was recorded as a further indicator of the extent of senescence. The data are shown as means ± SEM derived from six different individuals. The p values were derived from Student's *t*-test analysis (two-tailed) of the means of the indicated genotypes (*, significant at $p < 0.05$; **, significant at $p < 0.01$; ***, significant at $p < 0.001$). The wild type was used as the reference group for the statistical analysis.

(D) Analysis of leaf senescence in different *spl1* mutant alleles. Rosette leaves of 28-day-old plants were induced to senesce by covering with aluminium foil for 5 days. The leaves were excised at the end of the dark treatment and photographed. Control leaves were left uncovered. Multiple leaves from three plants per genotype per condition were analysed, and representative examples are shown.

(E) Gene expression analysis of *SP1*, *SPL1* and *SPL2*. The data were retrieved using the Genevestigator V3 analysis tool. The data shown are means ± SEM. The total number of samples used to derive each data point is indicated. Stages of development, from left to right, are defined as follows: germinating seed; seedling; rosette; developed rosette; bolting; young flower; developed flower; flower and siliques; mature siliques; and senescence. Typical ranges of low, medium and high expression are shown; medium is defined as the interquartile range (IQR).

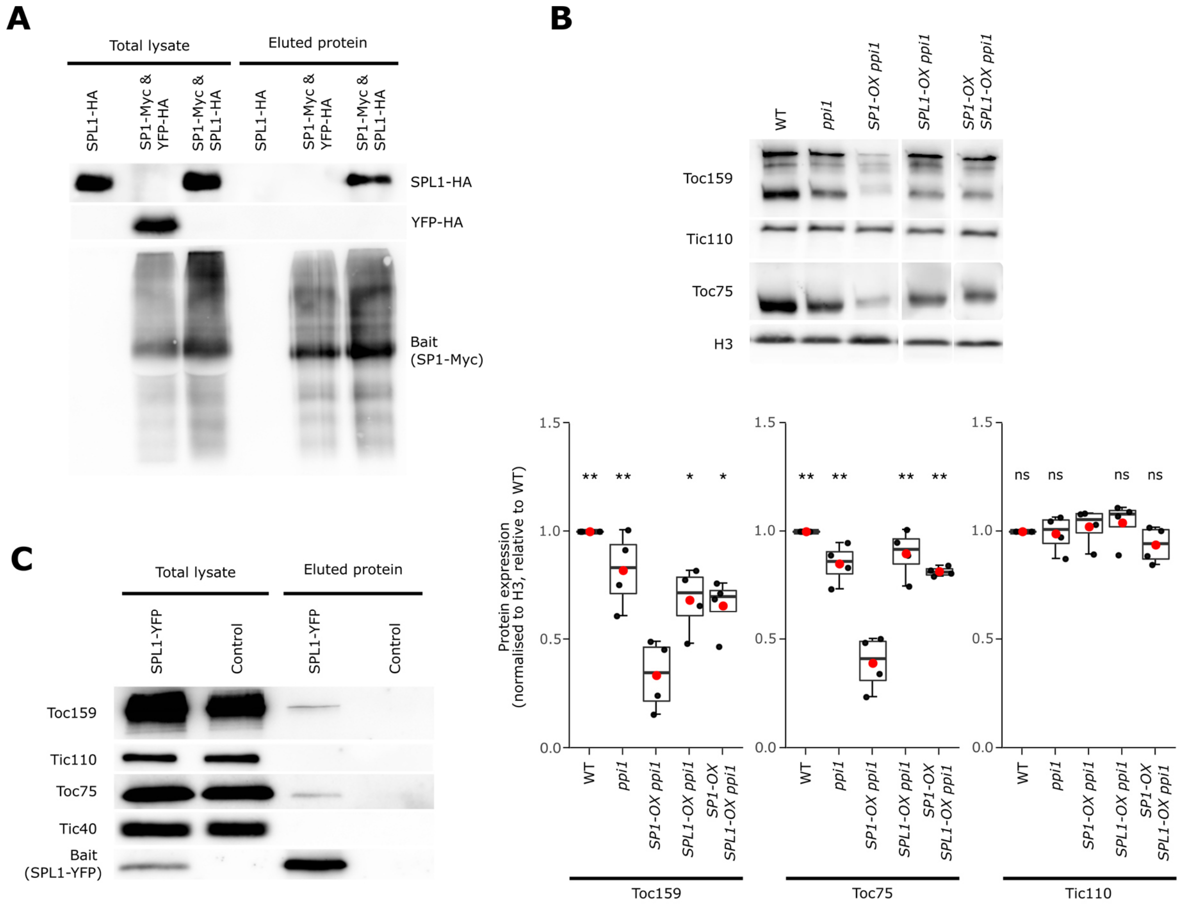

Figure S4. Interaction analyses of SPL1 and effects on the chloroplast protein import machinery, related to Figure 4.

(A) Immunoprecipitation analysis of physical interaction between SPL1 and SP1. The SPL1 and SP1 proteins were transiently co-expressed as HA- and Myc-tagged fusions, respectively, in *Arabidopsis* protoplasts; as a negative control, HA-tagged YFP was used. The cells were analysed by immunoprecipitation using anti-Myc antibody. Total lysate and eluted protein samples were analysed by immunoblotting using antibodies against: the Myc tag, to verify the enrichment of SP1-Myc bait protein; and the HA tag, to assess for interaction of SPL1-HA, or the YFP-HA negative control, with SP1-Myc.

(B) Immunoblot analysis of TOC protein accumulation in *SP1/SPL1-OX* plants. Total protein samples extracted from 28-day-old seedlings of the indicated genotypes were analysed by immunoblotting using a range of different TOC and TIC antibodies. Bands from four independent experiments were quantified and the data are shown as boxplots. The central box represents values between the first and the third quartiles, and the middle line represents the median value. The whiskers extend to the minimum and maximum values. The red circles indicate the means, and the black circles show the individual data points. The p values were derived from Student's *t*-test (two-tailed) of the means of the indicated genotypes (ns, not significant; **, significant at $p < 0.01$). The *SP1-OX ppi1* genotype was used as the reference group for the statistical analysis.

(C) Immunoprecipitation analysis of physical interaction between SPL1 and TOC proteins. The SPL1 protein was transiently expressed as a YFP fusion in *Arabidopsis* protoplasts, and employed as an immunoprecipitation bait. A sample lacking a fusion protein bait was used as a negative control, to confirm the specificity of the detected interactions. Total lysate and eluted protein samples were analysed by immunoblotting using antibodies against: the YFP

tag (anti-GFP was employed), to verify the enrichment of the fusion protein bait; TOC components, to assess for CHLORAD substrate interactions; and TIC components, to confirm the specificity of the detected TOC interactions.

Primer name	Sequence (5' to 3')*	Used to generate…
SPL2-cDNA-F	AAAAAGCAGGCTCCACTTGTCCGTGTGACCG	… full-length *SPL2* CDS for OX analysis
SPL2-cDNA-R	AGAAAGCTGGGTTATCGGTTACAAAATTTCTTCC	
SPL2amR1-I miR-s	GATACTAAGAGTAATATACGCGCTCTCTCTTTTGTATTCC	… *SPL2* amiRNA for KD analysis
SPL2amR1-II miR-a	GAGCGCGTATATTACTCTTAGTATCAAAGAGAATCAATGA	
SPL2amR1-III miR*s	GAGCACGTATATTACACTTAGTTTCACAGGTCGTGATATG	
SPL2amR1-IV miR*a	GAAACTAAGTGTAATATACGTGCTCTACATATATATTCCT	
SPL2-CDS-F	AAAAAGCAGGCTCCATGTCCTCGCCGGAGCGTG	… *SPL2* CDS for YFP fusion analysis
SPL2-nonstop-R	AGAAAGCTGGGTTAGAGTAATATACACGCATAG	
eIF4e1-F	AAACAATGGCGGTAGAAGACACTC	… RT-PCR data (note: for *SPL2* semi-quantitative analysis, primers SPL2-CDS-F and SPL2-cyt-R were used; for quantitative analysis, primers with the Q name prefix were used)
eIF4e1-R	AAGATTTGAGAGGTTTCAAGCGGTGTAAG	
Q-SPL2-F	ATGACCAAGGACAAGATGA	
Q-SPL2-R	ATGCCAACAGACACAATG	
Q-Actin2-F	TCAGATGCCCAGAAGTCTTGTTCC	
Q-Actin2-R	CCGTACAGATCCTTCCTGATATCC	
SPL1-cyt-F	GGGGACAAGTTTGTACAAAAAAGCAGGCTCCGTGATTGAATATATTCTA	… GST-SPL1cyt fusion for bacterial expression
SPL1-stop-R	GGGGACCACTTTGTACAAGAAAGCTGGGTTTCAATGGCGGTAAATTTTC	
SPL2-cyt-F	GGGGACAAGTTTGTACAAAAAAGCAGGCTCCGCTGCTGTCAGGACCTGGAA	… GST-SPL2cyt fusion for bacterial expression
SPL2-cyt-R	AGAAAGCTGGGTTCTAAGAGTAATATACACGCATAGAT	
SPL1-H308Y-F	GAGTGTGGTTATATGTGCTG	… SPL1-H308Y RING point mutation
SPL1-H308Y-R	CAGCACATATAACCACACTC	
SPL2-H348Y-F	CCTGCGTTTATTCCCTGTGGATATGTAGTATG	… SPL2-H348Y RING point mutation
SPL2-H348Y-R	CACATCGCCTGCAACATACTACATATCCACAG	

Table S1. Oligonucleotides used in this study, related to STAR Methods.

*Nucleotides shown in red correspond to linker sequences or mutation sites.
